# Portable Spatially Offset Raman Spectroscopy for Rapid Detection of Methanol and Ethanol in Pisco Through Sealed Containers

**DOI:** 10.3390/molecules31122120

**Published:** 2026-06-16

**Authors:** Yalan Wu, Beatriz Hatta-Sakoda, Victor Hugo Toledo-Herrera, Claudia Gastelumendi, Luis E. Rodríguez-Saona

**Affiliations:** 1 Department of Food Science and Technology, The Ohio State University, 110 Parker Food Science and Technology Building, 2015 Fyffe Road, Columbus, OH 43210, USA; wuyalan5671@gmail.com; 2 Food Technology Department, Faculty of Food Industries, Universidad Nacional Agraria La Molina, Lima 15024, Peru; bhs@lamolina.edu.pe; 3 Bodega San Nicolas, 1S 2014, Pisco 11600, Peru; victorhugoth@gmail.com (V.H.T.-H.); claudia.gastelumendi@bodegasannicolas.com (C.G.)

**Keywords:** Peruvian pisco, SORS Raman, methanol, ethanol

## Abstract

In this study, we introduced a portable spatially offset Raman spectroscopy (SORS) system that permits rapid, non-destructive acquisition of Raman spectra from bottled Pisco spirits, providing a practical and non-invasive solution for in situ quality control. Pisco, a Peruvian distilled spirit, was selected as a case study because methanol occurs naturally during fermentation and distillation, and the product is susceptible to adulteration. Methanol and ethanol levels in Pisco were determined using gas chromatography (GC–FID). Methanol levels in 94 authentic Pisco samples ranged from 7.4 to 67 mg/100 mL, remaining below the regulatory limits established for fruit brandies. For pure Pisco samples, the handheld SORS device demonstrated strong predictive performance for determining methanol (SEP = 0.003%, Rpre = 0.92) and ethanol (SEP = 1.25%, Rpre = 0.98) content. To further assess model applicability across a broader methanol concentration range, randomly selected Pisco samples were fortified with methanol (0.11–9.85%), resulting in a prediction model with excellent performance for methanol quantification (SEP = 0.17%; Rpre = 0.995). Overall, the SORS-based approach showed robust analytical capability, underscoring its potential as a non-contact, non-destructive technique for rapid quantification of methanol and ethanol in sealed glass containers.

## 1. Introduction

Globally, adulterated and counterfeit alcoholic spirits pose substantial risks to economic stability, labor conditions, and public health [1]. The World Health Organization (WHO) suggests that approximately 25% of spirits consumed worldwide may be counterfeit [2]. Common fraudulent practices in the distilled spirits industry include dilution with water to increase volume or substitution of ethanol with cheaper alcohols such as methanol [3] to artificially boost apparent alcohol strength [4]. The latter is responsible for most methanol poisoning incidents reported globally [5]. From 1998 to 2026, methanol poisoning affected 47,500 people and caused 14,700 fatalities worldwide [6], with Asia having the highest prevalence of methanol poisoning with outbreaks occurring in Indonesia, India, Cambodia, Vietnam, and the Philippines [6].

Ingestion over 2% (*v*/*v*) by volume of methanol in 40% alcohol by volume (ABV) drinks leads to irreversible tissue damage, especially to the eyes and nervous system, and even death [7,8]. This damage is attributed to metabolization of methanol to toxic formic acid and formaldehyde [9]. In the United States, maximum allowable methanol content in alcoholic beverages is restricted to 0.35% [10]. Consequently, proving that a suspect spirit is adulterated often requires an analysis of its chemical composition to demonstrate that it does not match the labeled alcohol content or to indicate the presence of contamination [11]. Ideally, such testing would be performed on-site with the product still sealed in its original packaging, and testing would be feasible at any point within a geographically distributed supply chain.

Chromatographic approaches are generally considered the reference standard for methanol determination in spirits [12], whereas ethanol content is commonly measured using densitometry [13]. Although these techniques provide accuracy, they typically require destructive sampling, are most often performed in laboratory environments, and depend on trained personnel and specialized equipment [14]. As a result, they are costly and are often inaccessible in low-resource settings, including many regions where methanol-poisoning outbreaks are most frequent [15]. Portable devices and headspace-sensing approaches have emerged as options for field screening [16,17,18]. For example, a pocket-sized sensor–smartphone platform for on-demand headspace analysis has been evaluated for detecting methanol-adulterated beverages [19], with reported capability to identify hazardous adulteration above 2% *v*/*v*. Another system integrating a chemoresistive metal-oxide gas sensor with a separation column has been developed for rapid on-site testing and reports detection of methanol down to 0.01% *v*/*v* within minutes [20]. However, these approaches still require opening the container and therefore cannot preserve the integrity of the original packaging. In practice, they remain destructive tests and yield results only after sampling has occurred. There is a clear need for simple, intact, rapid screening tools that could be used by first responders or even non-specialists to help spirits inspection.

Raman spectroscopy is widely regarded as an attractive “molecular fingerprinting” approach across many application areas [21,22]. It offers several practical advantages, including the availability of portable [23,24], rugged instruments suitable for field use [25], minimal or no sample preparation, and non-contact, non-destructive measurement [26,27]. In the context of alcoholic beverage analysis, Raman methods investigated to date include conventional Raman spectroscopy [28,29] as well as the more recently developed spatially offset Raman spectroscopy (SORS) [30,31]. For this study, Pisco, a distinctive grape-based brandy recognized as Peru’s emblematic national spirit, was selected. Pisco has been associated with methanol adulteration, resulting in severe injuries and deaths [32]. It is important to note that trace amounts of methanol are naturally formed during fermentation and are generally present at levels considered safe [33]. Pisco holds a Protected Denomination of Origin (PDO), which imposes strict production requirements for the final product: ethanol content has to fall to 38–48% ABV, and methanol must not exceed 100 mg/100 mL for non-aromatic grape Pisco or 150 mg/100 mL for aromatic grape Pisco [34]. Determination of ethanol and methanol levels in Pisco is fundamental for compliance with customs and excise legislation [35].

We summarized Raman spectroscopy-based studies from the past two decades that investigated ethanol and/or methanol screening in distilled spirits in Table 1. Earlier studies (2012–2016) were primarily laboratory bench-top investigations that required transferring samples into small glass vials prior to measurement [36,37,38]. Although they established proof-of-concept quantification, they relied on univariate calibration, in which concentration was estimated from the intensity or integrated area of one (or a few) characteristic Raman bands as the analyte concentration was varied. A major step toward real-world applicability occurred with the first through-container handheld Raman report in 2017 [28], which targeted ethanol and achieved a PLSR mean square error of 0.44%. After this milestone, handheld Raman combined with chemometrics became increasingly common, and many subsequent studies [39,40,41] prioritized authentication, counterfeit discrimination, and classification rather than rigorous simultaneous ethanol and methanol quantification monitoring. To date, only one study from our laboratory [42] has demonstrated validated quantitative analysis of naturally occurring methanol and ethanol in sealed Pisco containers using a 1064 nm conventional Raman device, illustrating that through-container quantification at realistic methanol levels is feasible.

With advances in deep Raman probing of diffusely scattering media, spatially offset Raman spectroscopy (SORS) shows promise in broadening the applicability of Raman spectroscopy to such sample types [43], enabling the interrogation of diffusely scattering packaging materials and containers [44]. It has shown early promises in spirits, allowing discrimination of 1%, 2%, and 3% methanol-spiked samples using PCA [30]. Subsequent SORS research on spirits has been limited to ethanol content prediction (PLSR: R^2^ = 0.93–0.97) and demonstrations of spectral similarity between SORS and conventional Raman for spirit measurements [45].

**Table 1:** Literature review of Raman spectroscopy-based ethanol and/or methanol screening studies from 2006 to 2026.

Year	Citation	Instrument	Laser (nm)	Methanol Concentration Range	Container/Barrier (Material, Color)	Model/Performance Metrics
2012	[37]	Raman Microscope (Deltanu Inc., Laramie, WY, USA)	785	Methanol: 0–10 M, Ethanol: 0–7 M	Glass vials	Univariate calibration, ethanol: R^2^ = 0.997, methanol: R^2^ = 0.998
2013	[38]	Raman Confocal microscope (Wotton-under-Edge, UK)	785	Methanol: 0.05–50%	Glass vials	Univariate calibration, methanol: R^2^ = 0.99
2016	[36]	Benchtop Raman (Deltanu Inc., Laramie, WY, USA)	785	Ethanol: 35.6–71.2%	Glass vials	Univariate calibration, R^2^ = 0.9997
2017	[28]	Benchtop Raman (Lafayette, CO, USA)	785	Ethanol: 40–60%	Whisky in clear bottle	PLSR: RMSE = 0.44%.
2017	[30]	Handheld SORS (Cobalt Light Systems, Oxfordshire, UK)	830	Methanol: 1–3%	Green, brown, and clear glass bottle	PCA cluster
2019	[41]	Handheld Raman (Snowy Range, Laramie, WY, USA)	1064	Methanol: 0–5%	Tinted bottles transferred to clear vials	PLSR, R = 0.93–0.97, strong fluorescence in colored tinted glass
2021	[42]	Portable Raman (Rigaku Corporation, Wilmington, MA, USA)	1064	Methanol: 1–283.6 ppm. Ethanol: 28.4–41.3%	Clear bottles	PLSR, methanol: R = 0.97, SEP = 9.8; ethanol: R = 0.94, SEP = 0.78
2022	[45]	Handheld SORS (Agilent Technologies, Santa Clara, CA, USA)	830	Ethanol: 30–70%	Clear glass bottle	PLSR: R = 0.971, SEV: 3.14%
2023	[40]	Self-developed conventional Raman system	532	Methanol: 0–200 g/L; Ethanol: 20–60%	Glass vial	PCA cluster
2024	[39]	Benchtop Raman (Logan, UT, USA)	785; 532	Methanol: 0–3%, Ethanol: 40–63%	Vials, through bottle, only evaluated for brand identify	PLSR, samples in vials, ethanol: R^2^ = 0.99, RMSEP = 0.25; methanol: R^2^ = 0.99, RMSEP = 0.05%.

Despite leveraging the container-suppression capabilities of SORS and benefiting from recent advances in spatial offsets and beam-shaping geometries [46], there have been no reports demonstrating quantitative SORS-based screening of naturally occurring, ultra-low methanol concentrations in sealed distilled spirits, particularly in the presence of challenging container interferences. This gap is especially significant given our prior work ([41,42]), which established the feasibility of through-container quantification of methanol (1–284 ppm) in Pisco using a portable 1064 nm Raman system. However, that study was restricted to measurements through optically clear glass containers, where conventional backscattering Raman spectroscopy can still effectively probe the liquid contents with minimal interference.

In contrast, the present study addresses a substantially more demanding analytical scenario by extending quantitative methanol detection to both clear and frosted glass containers using spatially offset Raman spectroscopy (SORS). Frosted glass presents a significantly greater optical barrier due to its strong diffuse scattering and dominant surface Raman contributions, which obscure subsurface signals and preclude accurate quantification using standard backscattering Raman approaches, including those employed in our previous work.

The objective of this study was to develop a rapid, non-destructive, and field-deployable SORS-based method capable of quantifying methanol and ethanol in Pisco directly through sealed glass containers, encompassing both optically clear and optically challenging frosted formats. Two practical and analytically distinct scenarios were addressed: (a) accurate determination of ethanol content and naturally occurring trace levels of methanol in authentic Pisco without opening the bottle, and (b) rapid, through-container detection of illicitly added methanol indicative of counterfeit or adulterated products. By explicitly addressing both container types, the proposed SORS approach establishes a more universally applicable solution for Pisco packaging conditions. Furthermore, targeting methanol alongside ethanol, and enabling quantification across both clear and highly scattering frosted containers, this work aims to deliver a reliable analytical tool for routine quality assessment as well as the detection of hazardous adulteration in commercially sealed Pisco spirits.

## 2. Results and Discussion

### 2.1. Reference Value of Pisco Samples

As presented in Table 2, GC–FID analysis of ethanol content demonstrated substantial variation among Pisco samples obtained from both premium producers and small-scale local distilleries, spanning a range of 22.06–66.06% (*v*/*v*). Under Pisco Protected Designation of Origin, Pisco is required to have an alcohol content falling between 38% and 48% ABV, and the addition of water after distillation is strictly prohibited [47]. Several samples in the present dataset failed to meet the minimum requirement of 38% ABV and would therefore be classified as “diluted Pisco.” Notably, the majority of these non-compliant samples originated from small-scale distilleries.

Methanol concentrations in original Pisco samples ranged from 7.37 to 67.26 mg/100 mL, with a mean value of 25.75 mg/100 mL. These values are well below the United States regulatory limit for methanol level in distilled spirits, which is 0.35% (*v*/*v*) at 40% alcohol [10]. All samples complied with the Pisco Protected Designation of Origin (PDO) regulations [34] and met the standards of identity for Pisco.

For the 50 methanol-spiked samples, final methanol concentrations ranged from 0.11% to 9.85% (*v*/*v*). These levels span values below the U.S. regulatory limit of 0.35% (*v*/*v*) at 40% alcohol as well as concentrations substantially exceeding typical naturally occurring levels, including those above 2% (*v*/*v*), thereby covering a broad range for method evaluation purposes.

### 2.2. Spectral Information of Pisco Samples

**Figure 1:**
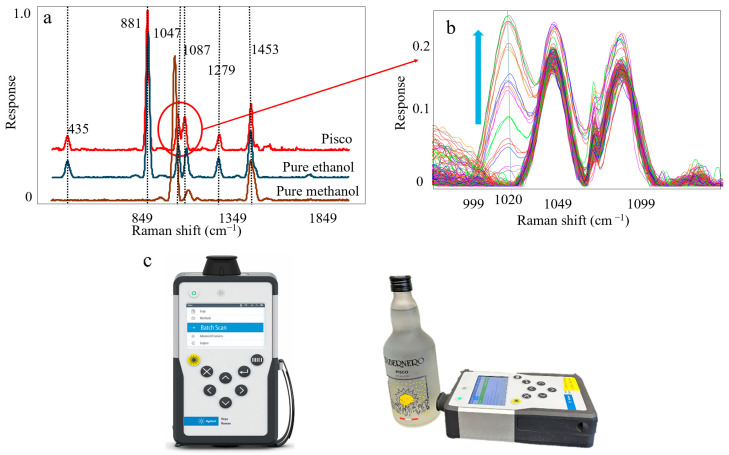
(**a**) SORS spectral of Pisco sample, pure ethanol, and methanol; (**b**) Increase in Raman intensity in the methanol spectral region with increasing methanol concentration; blue arrow shows the increase in methanol content led to an increase in the Raman intensity at 1020 cm^−1^; (**c**) Through-container measurement using portable Agilent Vaya SORS device.

Raman spectra of the samples displayed sharp, well-defined bands characteristic of polarizable functional groups, particularly symmetric vibrations of nonpolar moieties (Figure 1a). Pure ethanol and methanol spectrum were also acquired and overlaid in Figure 1a. The spectral profile of Pisco was consistent with previously reported findings and showed strong resemblance to that of pure ethanol [45]. Given that ethanol is the predominant component of Pisco aside from water, its characteristic Raman bands dominated the overall spectral profile. The dominant band at 881 cm^−1^ was assigned to the symmetric C–C–O stretching mode of ethanol [35]. A lower-intensity band observed at 435 cm^−1^ was associated with deformation vibrations of C–C–O and C–C bonds [42]. Bands appearing at 1047 cm^−1^ and 1087 cm^−1^ were attributed to C–C stretching and asymmetric C–C–O stretching modes, respectively [45]. The feature located at 1279 cm^−1^ was linked to the twisting mode of –CH_2_– groups, whereas the band at 1453 cm^−1^ was ascribed to the asymmetric bending vibration of –CH_3_ groups [36].

Figure 1b presents the overlaid spectra of authentic Pisco samples and methanol-spiked samples. A shoulder peak centered at 1020 cm^−1^ was identified as a critical marker for methanol quantification and was assigned to the C–O stretching vibration [41]. Notably, the intensity of this band increased progressively with rising methanol concentration, indicating its suitability for monitoring methanol levels.

### 2.3. Methanol and Ethanol Quantification in Pisco

Ethanol and methanol concentrations in Pisco were simultaneously predicted by correlating Raman spectra with GC–FID reference values by employing PLSR. Prior to the modeling, all samples were randomly assigned to calibration and external validation sets. Table 3 summarizes the performance of PLSR models. Inconsistency in sample numbers across analytes is attributable to the removal of outliers during model building. Mean-centering was applied to the calibration data prior to modeling. Outlier detection was conducted using studentized residual versus leverage diagnostic plots generated in Pirouette (Infometrix Inc.). The software determines critical limits based on an F-distribution at the 95% confidence level. Samples exceeding the corresponding thresholds for both studentized residuals and leverage were designated as potential outliers. This criterion is equivalent to applying statistically defined cutoffs for both response deviation and influence and was applied uniformly and without modification across all models. For methanol prediction, optimal model performance was achieved using spectral smoothing with a Savitzky–Golay polynomial filter (25-point window) followed by second-derivative transformation (35-point window). The ethanol prediction model showed the best performance when normalization was combined with Savitzky–Golay smoothing (35-point window) as spectra transformation method. The selection of Savitzky–Golay (SG) smoothing (25-point window) and second-derivative (35-point window) parameters were selected as a result of systematic optimization aimed at balancing noise reduction and preservation of chemically meaningful spectral information. A wide range of window sizes were evaluated during preliminary model development, and the chosen parameters consistently yielded the lowest RMSECV while maximizing model robustness and predictive performance. While the selected window sizes may appear relatively large, this choice is justified by the characteristics of the spectral data. Specifically, the spectra exhibit modest noise, and aggressive smoothing was not necessary to improve signal-to-noise ratio. Importantly, the SG filter preserved the shape and position of spectral features. We verified that these preprocessing settings do not distort or obscure the key analytical features relevant to methanol quantification. In particular, the C–O stretching band near 1020 cm^−1^—central to the predictive model—remains well-resolved and stable after preprocessing. The second-derivative transformation further enhances spectral resolution by reducing baseline variation and resolving overlapping bands, which supports more robust regression modeling. The selected SG window sizes represent an optimal trade-off: smaller windows resulted in insufficient noise suppression and reduced predictive accuracy, while larger windows began to attenuate relevant spectral variance. Therefore, the chosen parameters reflect the best compromise between noise reduction, feature preservation, and predictive capability.

The PLSR models achieved improved performance after excluding low and high wavenumber regions characterized by unstable and noisy baselines. Restricting the analysis to selected spectral intervals enhanced predictive accuracy compared with using the full spectral range, as this approach removed variables that were uninformative, noisy, or otherwise inconsistent [48]. During model optimization, the number of latent variables was selected based on the minimum standard error of cross-validation (SECV). The optimal models required five factors for ethanol and methanol, providing sufficient explanatory power to capture relevant variance while avoiding overfitting or loss of essential information [49]. As summarized in Table 3, both models exhibited strong predictive performance, with calibration correlation coefficients (Rcv) of 0.98 and 0.90 for ethanol and methanol, respectively. The cross-validation errors (RMSECV) were low, at 1.36% for ethanol and 2.72 mg/100 mL for methanol, indicating good accuracy and robustness of the developed models. The RMSEP of approximately 3 mg/100 mL for methanol should be evaluated in the context of both regulatory thresholds and the intended analytical application. In the case of Pisco PDO, the regulatory limit for methanol is on the order of 100–150 mg/100 mL, meaning that the observed prediction error corresponds to less than 3% of the lower specification limit. From an analytical perspective, this represents a relatively small uncertainty when compared to the concentration range of interest.

**Figure 2:**
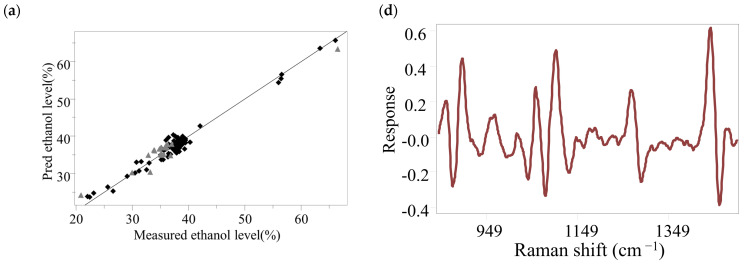

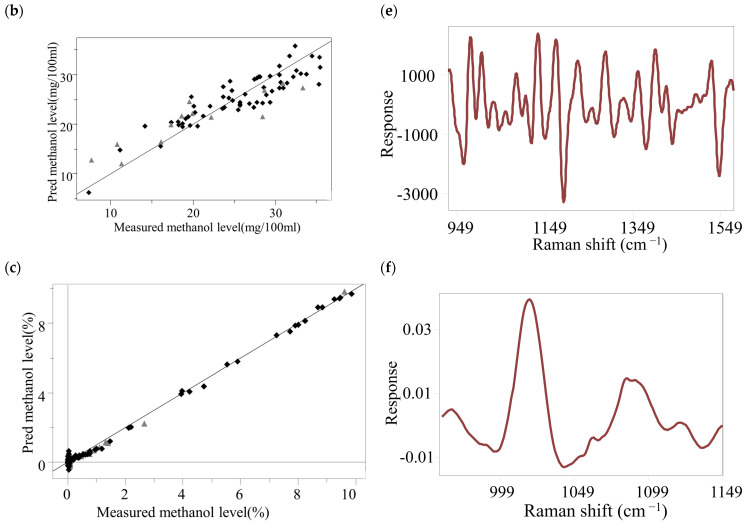
(**a**–**c**) Regression plots for PLSR prediction models. The black square shape scatters are the calibration set; the grey diamond-shape scatters are the validation set. (**d**–**f**) the regression vectors for each of the predictive models.

The PLSR regression coefficients and loading vectors are presented in Figure 2. In the regression vectors, positive and negative peaks indicate positive and negative correlations with the predicted variable, respectively, whereas values near zero denote negligible contribution. For ethanol prediction, the most influential spectral regions identified from the regression vector spanned 849–1479 cm^−1^ (Figure 2b). These regions correspond to vibrational modes associated with ethanol, including CH_3_ and CH_2_ bending, C–O stretching, and CH_3_ rocking vibrations, consistent with previous reports [35]. The regression vector for methanol (Figure 2d) exhibited strong contributions within the 940–1570 cm^−1^ range. In particular, bands between 980 and 1033 cm^−1^ were attributed to C–O stretching vibrations of methanol, while features in the 1437–1529 cm^−1^ region were associated with C–H asymmetric deformation modes [50]. These characteristic bands played a key role in the quantitative prediction of methanol content.

The predictive performance of the developed models, evaluated using the independent external validation subset, is summarized in Table 3. In all PLSR models, the leave-one-out cross-validation outcomes showed close correspondence with the external validation statistics, indicating robust alignment between internally and externally derived performance measures. As shown in the regression curves in Figure 2, the calibration and validation sets covered comparable concentration ranges and exhibited a strong linear relationship between predicted and reference values. The predicted results derived from the Raman spectra showed good agreement with the corresponding reference measurements for ethanol and methanol in Pisco samples, confirming the reliability of the proposed analytical approach.

### 2.4. Prediction of Methanol Level Includes Spiked Sample

PLSR was also applied to develop a comprehensive predictive model for methanol quantification that includes both methanol-spiked samples and authentic Pisco samples. The model was constructed following the same procedures described in Section 3.3. A cross-validated calibration model was established using measurements obtained from genuine Pisco bottles, and model robustness was evaluated against an independent external test set. A summary of model performance across both calibration and validation is provided in Table 3. The cross-validated calibration model was developed using normalization and Savitzky–Golay smoothing (25-point window) as the spectra transformation method. Three latent variables were selected, resulting in a cross-validation correlation coefficient (Rcv) of 1.00 and a standard error of cross-validation (SECV) of 0.23%. The model’s robustness was supported by comparable values between calibration and validation datasets, with a Rpre of 1.00 and a RMSEP of 0.17%, closely matching the SECV.

The PLSR correlation plots and regression vectors are presented in Figure 2. The data points are closely distributed along the 1:1 line, indicating excellent alignment between predicted and measured values. The regression vector plot highlights a strong contribution from the band at 1020 cm^−1^, corresponding to the C–O stretching vibration in methanol, which plays a critical role in model development. Additionally, the band near 1079 cm^−1^, attributed to CH_3_ rocking vibrations in methanol, also contributes to the predictive model.

The differences in ordinate scale observed across regression vectors for different analytes (Figure 2d–f) are an expected outcome of the underlying chemometric framework and do not reflect inconsistencies in model quality or interpretability. In PLSR, the absolute magnitude of regression coefficients is inherently dependent on several factors, including spectral preprocessing, variable scaling (e.g., mean-centering, normalization, derivative transformations), and the variance structure of both predictor and response variables. Furthermore, the magnitude of regression coefficients is influenced by the scaling of the response variable (analyte concentration range and variance). Models built for analytes with narrower concentration ranges or lower variance may naturally produce coefficients of different magnitude compared to those with broader dynamic ranges, even when the underlying spectral relationships are equally strong. The key information in regression vectors in PLSR lies in the positions of bands and troughs and their correspondence to chemically meaningful absorption bands. These patterns provide insight into which regions of the spectrum contribute to the model, regardless of the overall scaling of the coefficients. Therefore, the observed differences in regression vector scale are a normal and expected consequence of preprocessing choices and data scaling, and do not impede interpretation.

Practical field use of handheld SORS devices may involve temperature fluctuations, variations in bottle curvature, label occlusion, and differences in glass color (clear vs. frosted). Our study accounted for variation in bottle geometry by deliberately collecting spectra from multiple regions on each container, including two locations along the bottle body and one at the neck. These positions inherently capture differences in local curvature and optical path geometry. The consistent predictive performance observed across all measurement locations indicates that the SORS approach—and the developed models—are robust to curvature-related variability typical of commercial Pisco bottles.

While controlled temperature studies were not conducted, the Agilent Vaya platform is specifically engineered for field use, with operational stability across typical ambient conditions encountered in on-site inspections. Nevertheless, extreme environmental conditions could introduce additional variability and represent a useful direction for future validation. Label interference was intentionally minimized in this study to isolate the intrinsic performance of the SORS method for through-container analysis. As such, spectra were collected from label-free regions of the bottles. Label occlusion is an important practical consideration for future research, as printed inks and adhesives can introduce spectral interference or attenuate Raman signals. Finally, our study included both clear and frosted glass bottles, which are representative of the majority of commercial Pisco packaging. The comparable predictive performance obtained across these two container types demonstrates that the method is robust to differences in light scattering and transmission associated with these materials. Tinted or highly colored glass was not evaluated, as it is uncommon in Pisco products; however, such materials could introduce additional optical attenuation or fluorescence effects.

## 3. Materials and Methods

### 3.1. Sample Collection and Preparation

Ninety-four samples were sourced through a collaborative arrangement with Pisco 1615 Inc. (Pisco, Peru) and the Universidad Nacional Agraria La Molina (Peru). The samples were obtained from seven distilleries in the Ica region, with collection taking place across grape harvesting periods from 2023 to 2025. The sample set comprises Pisco derived from multiple grape cultivars, namely Italia (*N* = 13), Moscatel (*N* = 4), Albilla (*N* = 10), Quebranta (*N* = 27), Torontel (*N* = 11), and mixed cultivar blends (*N* = 29). Among the 94 samples, four correspond to the “head” fraction of the distillation process, which typically exhibits a higher concentration of volatile compounds relative to commercially available Pisco products. All samples were packaged in 100 mL clear or frosted glass bottles and kept sealed in their original containers for Raman spectral measurement.

Methanol levels in the samples were consistently low and narrowly distributed, with values ranging from 7.37 to 66.1 mg/100 mL (standard deviation = 8.33). A spiking strategy was employed to extend the concentration range to levels representative of real-world methanol adulteration scenarios and to enhance the robustness of the predictive model. The concentrations achieved are in line with those documented in cases of economically motivated food fraud. Authentic Pisco distillate matrices were used as spiking media in place of simplified solvent systems to reduce potential matrix effects. Twenty samples were randomly selected and fortified manually with analytical-grade methanol. A single spiking event was applied to each 100 mL sample at an assigned quantity ranging from 90 to 9700 mg, generating 50 spiked samples in total. The fortification level was varied across preparations to ensure a unique methanol concentration in each spiked sample. Exact methanol concentrations were subsequently determined using reference methods as described in Section 2.2.

### 3.2. Determination of Ethanol and Methanol Concentration

Reference values were determined following a previously reported method [51] with modifications. Triplicate analyses were conducted for all samples, with mean values taken for subsequent data analysis. Methanol and ethanol concentrations were determined by an Agilent Technologies 6890 gas chromatograph (GC) coupled with a flame ionization detector (FID) (Santa Clara, CA, USA). Separation was achieved on an HP-FFAP column (25 m × 320 µm internal diameter × 0.5 µm film thickness; Agilent Technologies, Santa Clara, CA, USA). Sample injection was performed in split mode (15:1), with a 1 µL aliquot introduced at a constant carrier gas flow rate of 24.3 mL/min and an injector temperature of 250 °C. The oven was programmed as follows: 40 °C held for 4 min; increased at 5 °C/min to 100 °C; then at 10 °C/min to 160 °C; and finally at 55 °C/min to 220 °C, maintained for 2 min. Two separated methanol standard curves were made using 99.9% pure methanol (Sigma-Aldrich, St. Louis, MO, USA) dissolved in a 40% (*v*/*v*) ethanol/water mixture, with concentration ranges of 0.75–153.4 mg/100 mL and 0.06–12.5% (*v*/*v*). A 70% (*v*/*v*) ethanol stock solution was prepared by adding 70.0 mL of pure ethanol reagent into a 100 mL volumetric flask and adjusting to volume with deionized water. A series of ethanol working standards ranging from 10 to 70% (*v*/*v*) were subsequently obtained through successive dilution of the stock solution using deionized water.

### 3.3. Through-Container Measurement of SORS Raman Spectra

Spectral acquisition was performed using a handheld Vaya Raman SORS instrument (Agilent Technologies, Santa Clara, CA, USA) using an excitation wavelength of 830 nm laser and an output power of 450 mW. Raman spectra were recorded over the range of 350–2000 cm^−1^, with a spectral resolution of 12–20 cm^−1^. Each measurement consisted of the average of 10 scans acquired through the container in under 2 min under ambient light conditions. Spectral acquisitions of empty containers were also performed to support the instrument’s internal signal separation algorithm. These empty-container spectra were not used as independent inputs in the PLSR models, nor were they manually subtracted during post-processing. Instead, the Vaya instrument uses them within its built-in scaled-subtraction procedure to minimize the spectral contribution of the container material, yielding the final processed SORS spectrum in which the container contribution has been substantially removed. The Vaya instrument yields three output files for each measurement: (i) a zero spectrum (Raman signal captured in the absence of offset), (ii) an offset spectrum (Raman signal captured with a 7 mm spatial offset), and (iii) a processed spectrum (baseline-corrected and 0–1 normalized following scaled subtraction executed by the instrument software). This third file will hereafter be designated as the final-SORS throughout the present study.

For each sample, two spectra were collected from randomly selected positions along the bottle body and one from the neck region; the three replicate spectra obtained were then averaged prior to chemometric analysis. Thus, only a single averaged spectrum per sample entered the calibration or validation sets, thereby completely eliminating the possibility of spectral replication bias or artificial inflation of model performance due to duplicated measurements.

### 3.4. Chemometrics Analysis

Spectral data were analyzed using multivariate statistical software (Pirouette^®^ version 5.0, Infometrix Inc., Woodville, WA, USA). Quantitative analysis in Pisco was accomplished through partial least squares regression (PLSR), whereby spectral data were related to reference measurements via calibration modeling [48,49]. The full dataset was randomly partitioned into a calibration subset (80%) used for model development and an independent external test subset (remaining 20%) reserved for evaluating predictive performance.

Before PLSR model construction, spectral data underwent preprocessing to improve model robustness and minimize systematic variability. Multiple commonly used preprocessing approaches (including smoothing, normalization, and derivative treatments) were tested for each analyte, and the reported combinations were selected because they yielded the lowest RMSECV values while preserving chemically meaningful spectral features relevant to quantification. Normalization was performed using the “Normalize to: 100” function, which scales each spectrum so that its maximum intensity equals 100 and all remaining intensities are scaled proportionally. Savitzky–Golay (SG) smoothing was applied to reduce high-frequency noise while preserving band shape and spectral resolution. Applying the second derivative (after SG smoothing) enhances band resolution, corrects baseline offsets, and improves discrimination of overlapping peaks, thereby facilitating more accurate quantitative analysis. Each spectrum was mean-centered and subsequently transformed by applying normalization, smoothing with a window size of 25 points or second-derivative Savitzky–Golay filter with a window size 35 points. The use of different preprocessing strategies for methanol and ethanol reflects inherent differences in their spectral characteristics and analytical context, rather than inconsistent methodology. Ethanol is present at relatively high concentrations and exhibits strong, well-defined Raman bands; thus, normalization combined with smoothing was sufficient to stabilize signal intensity and reduce noise without distorting peak shapes. In contrast, methanol is present at trace levels with comparatively weaker spectral contributions. For this reason, Savitzky–Golay smoothing followed by second-derivative processing was necessary to enhance subtle spectral features and improve resolution of overlapping bands, thereby improving quantification performance. The same objective selection criterion (minimization of RMSECV) was applied consistently across both analytes. To avoid any risk of overfitting through preprocessing selection, all comparisons were performed using internal cross-validation within the calibration set only, and preprocessing choices were fixed prior to evaluation on the independent validation set.

Model performance was assessed using the cross-validation correlation coefficient (Rcv), the root mean square error of cross-validation (RMSECV), the prediction correlation coefficient (Rpre), and the root mean square error of prediction (RMSEP) [49,50]. Potential outliers were identified through plots of studentized residuals versus leverage values [50,52,53]. The appropriate number of latent variables for each PLSR model was determined by selecting the value that minimized the RMSECV [54]. The RMSEP quantifies the prediction error associated with independent external samples. Samples with large residuals, atypical patterns, or high leverage were treated as outliers and removed from the final models to improve robustness and reliability [55].

## 4. Conclusions

This study demonstrates that a portable spatially offset Raman spectroscopy device integrated with PLSR enables rapid and reliable quantification of ethanol and methanol in Pisco through sealed containers. The method requires no sample transferring and delivers results within approximately one minute. To our knowledge, this is the first report describing quantitative monitoring of ethanol and methanol concentrations in distilled spirits through intact packaging using a rapid, non-invasive SORS approach.

PLSR modeling exhibited strong linear regression, supporting the simultaneous prediction of ethanol and naturally occurring extremely low methanol concentrations for through-container quality control. To broaden the applicability of the methanol prediction model, methanol was deliberately spiked into authentic Pisco samples, thereby extending the calibration range and improving relevance to real-world scenarios, including methanol contamination or poisoning incidents. A unified calibration model was subsequently established by combining original and spiked samples. This model demonstrated great predictive capability, achieving a Rpre of 0.995 and a low RMSEP of 0.17%.

The SORS approach in this study shows strong potential for rapid, on-site screening of intact Pisco products, including detection of water dilution or methanol adulteration. The present study demonstrated successful through-container measurements using both clear and frosted glass bottles, which is challenging for conventional Raman spectroscopy due to strong surface scattering. However, the current study does not address the challenges associated with darker spirits or colored glass bottles as Pisco is typically colorless and packaged in clear or frosted containers. Investigating the performance of this technique under a wider range of real-world conditions represents an important direction for future research. Overall, this work highlights the promise of SORS spectroscopy for enhancing product safety and may stimulate further development of non-destructive, high-throughput analytical tools for quality control in the spirits industry. This study does not address the challenges associated with darker spirits or colored glass bottles, as Pisco is typically colorless and packaged in clear or frosted containers. Investigating the performance of this technique under more optically challenging conditions represents an important direction for future research. Overall, this work highlights the promise of SORS spectroscopy for enhancing product safety and may stimulate further development of non-destructive, high-throughput analytical tools for quality control in the spirits industry.

## Figures and Tables

**Table 2:** Descriptive statistics of ethanol and methanol concentrations in the analyzed samples as determined by GC–FID.

Parameter	N	Minimum	Maximum	Average	STD
Ethanol (%)	94	22.06	66.06	37.81	7.08
Methanol (mg/100 mL)-Pure Pisco	94	7.37	67.26	25.75	8.33
Methanol (%)-spiked	50	0.11	9.85	3.15	3.43

**Table 3:** Predictive performance of PLSR models for Pisco analysis using a portable SORS instrument.

Parameter	Calibration Model				External Validation Model			
	N	Factor	RMSECV	Rcv	N	Factor	RMSEP	Rpre
Ethanol (%)	73	5	1.36	0.98	19	5	1.25	0.98
Methanol (mg/100 mL)-pure Pisco	61	5	2.07	0.90	16	5	3	0.95
Methanol (%)-spiked	116	3	0.23	1.00	30	3	0.17	0.995

## Data Availability

The original contributions presented in this study are included in the article. Further inquiries can be directed to the corresponding author.
